# An Individual Participant Data Population Pharmacokinetic Meta-analysis of Drug-Drug Interactions between Lumefantrine and Commonly Used Antiretroviral Treatment

**DOI:** 10.1128/AAC.02394-19

**Published:** 2020-04-21

**Authors:** Jose Francis, Karen I. Barnes, Lesley Workman, Tamara Kredo, Lasse S. Vestergaard, Richard M. Hoglund, Pauline Byakika-Kibwika, Mohammed Lamorde, Stephen I. Walimbwa, Ifeyinwa Chijioke-Nwauche, Colin J. Sutherland, Concepta Merry, Kimberley K. Scarsi, Nyagonde Nyagonde, Martha M. Lemnge, Saye H. Khoo, Ib C. Bygbjerg, Sunil Parikh, Francesca T. Aweeka, Joel Tarning, Paolo Denti

**Affiliations:** a Division of Clinical Pharmacology, Department of Medicine, University of Cape Town, Cape Town, South Africa; b WorldWide Antimalarial Resistance Network (WWARN) Pharmacology/Southern African Regional Centre, University of Cape Town, Cape Town, South Africa; c Cochrane South Africa, South African Medical Research Council, Cape Town, South Africa; d Muheza District Hospital, Muheza, Tanzania; e Global Health Section, Department of Public Health, University of Copenhagen, Copenhagen, Denmark; f Department of Infectious Disease Epidemiology and Prevention, and Parasitology Laboratory, Statens Serum Institut, Copenhagen, Denmark; g Mahidol Oxford Tropical Medicine Research Unit, Faculty of Tropical Medicine, Mahidol University, Bangkok, Thailand; h Centre for Tropical Medicine and Global Health, Nuffield Department of Medicine, University of Oxford, Oxford, United Kingdom; i Department of Medicine, Makerere University, Kampala, Uganda; j Infectious Diseases Institute, Makerere University, Kampala, Uganda; k Department of Clinical Pharmacy and Management, Faculty of Pharmaceutical Sciences, University of Port Harcourt, Port Harcourt, Rivers State, Nigeria; l Department of Immunology and Infection, Faculty of Infectious and Tropical Diseases, London School of Hygiene & Tropical Medicine, London, United Kingdom; m Department of Clinical Medicine, Trinity College, Dublin, Ireland; n Department of Pharmacy Practice and Science, University of Nebraska Medical Center, Omaha, Nebraska, USA; o Tanga Research Centre, National Institute for Medical Research (NIMR), Tanga, Tanzania; p Department of Molecular and Clinical Pharmacology, University of Liverpool, Liverpool, United Kingdom; q Department of Epidemiology of Microbial Diseases, Yale School of Public Health, New Haven, Connecticut, USA; r Drug Research Unit, University of California, San Francisco, San Francisco, California, USA; s WorldWide Antimalarial Resistance Network (WWARN) Pharmacometrics/Asia Regional Centre, Mahidol University, Bangkok, Thailand

**Keywords:** uncomplicated malaria, lumefantrine, human immunodeficiency virus, drug-drug interactions, population pharmacokinetics

## Abstract

Treating malaria in HIV-coinfected individuals should consider potential drug-drug interactions. Artemether-lumefantrine is the most widely recommended treatment for uncomplicated malaria globally. Lumefantrine is metabolized by CYP3A4, an enzyme that commonly used antiretrovirals often induce or inhibit. A population pharmacokinetic meta-analysis was conducted using individual participant data from 10 studies with 6,100 lumefantrine concentrations from 793 nonpregnant adult participants (41% HIV-malaria-coinfected, 36% malaria-infected, 20% HIV-infected, and 3% healthy volunteers). Lumefantrine exposure increased 3.4-fold with coadministration of lopinavir-ritonavir-based antiretroviral therapy (ART), while it decreased by 47% with efavirenz-based ART and by 59% in the patients with rifampin-based antituberculosis treatment. Nevirapine- or dolutegravir-based ART and malaria or HIV infection were not associated with significant effects. Monte Carlo simulations showed that those on concomitant efavirenz or rifampin have 49% and 80% probability of day 7 concentrations <200 ng/ml, respectively, a threshold associated with an increased risk of treatment failure. The risk of achieving subtherapeutic concentrations increases with larger body weight. An extended 5-day and 6-day artemether-lumefantrine regimen is predicted to overcome these drug-drug interactions with efavirenz and rifampin, respectively.

## Abstract (precis)

Treating malaria in HIV-coinfected individuals should consider potential drug-drug interactions. Artemether-lumefantrine is the most widely recommended treatment for uncomplicated malaria globally. Lumefantrine is metabolized by CYP3A4, an enzyme that commonly used antiretrovirals often induce or inhibit. A population pharmacokinetic meta-analysis was conducted using individual participant data from 10 studies with 6,100 lumefantrine concentrations from 793 nonpregnant adult participants (41% HIV-malaria-coinfected, 36% malaria-infected, 20% HIV-infected, and 3% healthy volunteers).

## INTRODUCTION

Malaria is responsible for the heaviest burden of all parasitic infections, with an estimated 219 million cases and 435,000 deaths reported worldwide in 2017 (1). The vast majority of malaria deaths (∼93%) occurred in the sub-Saharan Africa region. Due to the substantial geographical overlap between malaria and HIV, many patients require concomitant treatment with both antimalarial drugs and antiretroviral drugs (ARVs). This creates a potential for drug-drug interactions, which may affect the antimalarial treatment outcome. Artemether-lumefantrine (AL) is the most widely used first-line treatment for uncomplicated falciparum malaria globally. Artemether is rapidly converted to its active metabolite dihydroartemisinin, which rapidly reduces the parasite biomass with a short terminal half-life of 1.5 to 2 h, whereas lumefantrine displays a longer terminal half-life of 3 to 5 days and is responsible for clearing the remaining parasites to prevent recrudescence (2). The absorption of lumefantrine is readily saturable (i.e., dose limited) and markedly affected by food, as coadministration of fat increases its absorption (2, 3). Lumefantrine is highly protein bound, mostly to high-density lipoproteins. It is mainly metabolized by CYP3A4 to desbutyl-lumefantrine, a compound that appears to be more active against malaria than lumefantrine. However, since systemic lumefantrine exposure is 85- to 300-fold higher than desbutyl-lumefantrine, lumefantrine is considered responsible for most antimalarial activity (4–6). Many of the first- and second-line ARVs currently used in developing countries affect the expression or activity of the CYP3A4 enzyme. Ritonavir is a potent inhibitor of CYP3A4, while efavirenz and nevirapine are both inducers, the latter with a weaker induction capacity than the former (7). Various studies have reported that lumefantrine exposure is significantly decreased when coadministered with efavirenz-based antiretroviral therapy (ART) and increased when given with lopinavir-ritonavir-based ART (8–12). Results are inconsistent in the few studies investigating the effect of nevirapine-based ART on lumefantrine exposure, showing increased, decreased, or no effect on lumefantrine exposure (13–18). Dolutegravir-based ART is being rapidly adopted as first-line HIV treatment; dolutegravir is an HIV-integrase inhibitor reported to have minimal effects on CYP3A4, and a study has shown that DTG-based ART did not alter lumefantrine exposure significantly (19, 20). Additionally, no information is available on whether malaria or HIV disease may affect lumefantrine pharmacokinetics.

The purpose of this meta-analysis was to pool available clinical data to characterize the effect of ARV drug-drug interactions with artemether-lumefantrine and to identify any other significant covariates affecting lumefantrine concentrations. This meta-analysis did not simply collate aggregate results from individual studies but jointly reanalyzed the individual participant data, using a population pharmacokinetic modeling approach. This technique can identify and quantify the different sources of variability in the data, thus separating the random unexplained differences between participants and studies from systematic effects, such as those associated with patient characteristics (e.g., weight or age), drug-drug interactions, and/or disease effects. With an increased and diverse study population and larger variability in treatment scenarios obtained when pooling individual participant data from different studies, it is possible to quantify the extent of drug-drug interactions and other covariates more robustly than in the individual primary studies.

## RESULTS

### Data summary.

A total of 16 artemether-lumefantrine pharmacokinetic studies were identified in a literature review and invited by WorldWide Antimalarial Resistance Network (WWARN) to contribute to the individual patient data (IPD) meta-analysis. Studies addressing the pharmacokinetics of lumefantrine in pregnant women and children were not included, as it was beyond the scope of this meta-analysis. WWARN received and curated data from 11 artemether-lumefantrine pharmacokinetic studies, 9 from Africa and 2 from the United States, but one U.S. study only contributed summary values (not individual patient data) and was excluded. Thus, the IPD meta-analysis consisted of 10 clinical studies with 793 nonpregnant adult participants and 6,100 measured lumefantrine concentrations. Out of these, the concentrations in 341 (5.59%) samples were below the lower limit of quantification (LLOQ). All participants were nonpregnant adults treated with artemether-lumefantrine (Coartem; Novartis Pharma AG). The distribution of participants and their demographic characteristics across different studies are presented in Table 1, while an overview of the studies included is provided in Table 2.

**TABLE 1:** Distribution of patients and their characteristics across the studies included in the analysis^*a*^

Patient group by study (reference) [study name]	No. of participants (no. of samples)	Infection status for:		ART treatment	Wt (median [range]) (kg)	Fat-free mass (median [range]) (kg)	Age (median [range]) (yr)	Sex (no. of male/no. of female)
		Malaria	HIV					
Kredo et al. (10, 15) [SEACAT 2.4.1 and 2.4.2]	55 (1,908)	Not infected	Positive		59.0 (45.5–88.0)	41.1 (31.3–59.7)	32 (19.6–60.9)	10/45
HIV^+^, malaria uninfected, not on ART	18 (569)	Not infected	Positive	None	58.0 (52.0–88.0)	37.8 (34.3–51.9)	27.6 (19.6–39.6)	1/17
HIV^+^, malaria uninfected, on NVP-based ART	18 (449)	Not infected	Positive	NVP	58.5 (45.5–80.0)	39.2 (31.3–59.6)	32.2 (28.1–60.9)	3/15
LPV-r phase 1 (single dose of AL)	18 (378)	Not infected	Positive	LPV-r	60.5 (46.0–85.0)	43.8 (31.9–52.5)	36.9 (28.1–44.6)	6/12
Phase 2 (multiple doses of AL)^*b*^	16 (512)	Not infected	Positive	LPV-r	62.0 (50–85.0)	44.5 (34.2–54.5)	37.4 (30.1–44.9)	5/11
InterACT study (53) [InterACT]	441 (1,749)	Infected	Positive and negative		53.0 (30.0–96.0)	38.6 (24.7–66.6)	38.0 (14.0–59.0)	252/189
Malaria infected	186 (684)	Infected	Negative	None	54.0 (30.0–92.0)	41.5 (27.9–66.6)	26.0 (14.0–58.0)	80/106
HIV^+^, malaria infected, not on ART	52 (190)	Infected	Positive	None	52.0 (34.0–81.0)	35.9 (24.7–59.6)	40.0 (17.0–59.0)	33/19
HIV^+^, malaria infected, on NVP-based ART	109 (476)	Infected	Positive	NVP	53.0 (33.0–96.0)	36.4 (24.9–60.7)	40.0 (15.0–54.0)	86/23
HIV^+^, malaria infected, on EFV-based ART	94 (399)	Infected	Positive	EFV	52.0 (39.0–86.0)	37.4 (27.3–56.8)	43.5 (20.0–59.0)	53/41
Byakika-Kibwika et al. (9, 17) [Uganda study 1 and study 2]	90 (1,324)	Not infected	Positive		60.5 (42.0–91.0)	39.51 (29.3–60.1)	36.0 (20.0–70.0)	24/66
Study 1								
HIV^+^, malaria uninfected, not on ART	13 (116)	Not infected	Positive	None	64.0 (50.0–81.0)	41.8 (34.1–51.9)	34.0 (24.0–51.0)	4/9
HIV^+^, malaria uninfected, on LPV-r-based ART	18 (170)	Not infected	Positive	LPV/r	64.5 (45.0–86.0)	45.5 (31.7–47.4)	37.5 (25.0–44.0)	8/10
Study 2								
Phase 1, HIV^+^, malaria uninfected, not on ART	59 (524)	Not infected	Positive	None	56.0 (42.0–91.0)	45.4 (36.5–57.9)	36.0 (20.0–70.0)	12/47
Phase 2a, HIV^+^, malaria uninfected, on NVP-based ART^*c*^	28 (249)	Not infected	Positive	NVP	54.5 (42.0–78.0)	44.6 (36.6–55.5)	33.5 (20.0–62.0)	1/27
Phase 2b, HIV^+^, malaria uninfected, on EFV-based ART	30 (265)	Not infected	Positive	EFV	62.0 (43.0–91.0)	48.7 (37.6–57.9)	38.0 (23.0–70.0)	11/19
Parikh et al. (14) [Nigeria study 1]	11 (164)	Not infected	Positive	NVP	66.0 (56.0–92.0)	44.3 (36.8–56.9)	37.0 (31.0–60.0)	2/9
HIV^+^, malaria uninfected, on NVP-based ART								
Chijioke-Nwauche et al. (18) [Nigeria study 2]^*d*^	167 (167)	Infected	Positive and negative		65.0 (25.0–97.0)	NA	36.0 (17.0–66.0)	20/147
HIV^−^, malaria infected, not on ART	99 (99)	Infected	Negative	None	65.0 (25.0–97.0)	NA	37.5 (17.0–49.5)	1/98
HIV^+^, malaria infected, on NVP-based ART	68 (68)	Infected	Positive	NVP	65.0 (40.0–92.0)	NA	36.0 (22.0–66.0)	19/49
German et al. (2009) (8) [U.S. healthy volunteer study]	10 (318)	Not infected	Negative		84.3 (55.8–104.7)	62.2 (38.1–74.6)	27.5 (21.0–45.0)	6/4
Phase 1, HIV^−^, malaria uninfected				None				
Phase 2, HIV^−^, malaria uninfected				LPV-r				
Lamorde et al. (29) [Uganda study 3]	5 (110)	Not infected	Negative	None	61.0 (41.5–69.0)	39.9 (30.3–48.6)	30.0 (23.0–50.0)	1/4
HIV^−^, malaria uninfected, on RIF-based antituberculosis treatment								
Walimbwa et al. (20) [Uganda study 4, healthy volunteer study]	14 (361)	Not infected	Negative		57.8 (45.0–76.0)	49.1 (32.3–56.9)	29.0 (19.0–32.0)	8/6
Phase 1, HIV^−^, malaria uninfected				None				
Phase 2, HIV^−^, malaria uninfected				DTG				

a HIV^+^, HIV positive; HIV^−^, HIV negative; NVP, nevirapine; LPV-r, lopinavir-ritonavir; EFV, efavirenz; RIF, rifampin; DTG, dolutegravir.

b 15 subjects were recruited in both phases.

c Only 58 patients’ data were available for phase 2 of Uganda study 2.

d The information on age was available for 76 participants, and the height measurement was missing for all the participants; therefore, the fat-free mass was not calculated.

**TABLE 2:** Summary of the study pharmacokinetic protocols

Study name (reference)	Country	Treatment (protocol)	Sampling schedule (protocol)	Sample collection type	Sample assay method (LLOQ [ng/ml])^*a*^
SEACAT 2.4.1 (15)	South Africa	480 mg Coartem twice daily for 3 days (0, 8, and 24 h, thereafter every 12 h) taken with 40 ml of soy milk (0.8 g fat) and, for all doses except the second, a meal containing 6 g of fat within 1 h of each dose	0, 0.5, 1, 1.5, 2, 3, 4, 5, 6, 8, 14, 24, 30, 36, 42, 48, 54, 60, 61.5, 62, 63, 64, 65, 66, 68, 70, 72, 96, 120, 144, 168, 336, and 504 h after the 1st dose	Plasma from venous blood	LC-MS/MS (20)
SEACAT 2.4.2 (10)	South Africa	Phase 1, 480 mg Coartem, single dose taken with 40 ml of soy milk (0.8 g fat) and a meal containing 6 g of fat within 1 h of dose administration	0, 0.5, 1, 1.5, 2, 3, 4, 5, 6, 8, 14, 24, 30, 36, 42, 48, 54, 60, 72, 96, 120, 144, 168, 336, and 504 h after the 1st dose^*b*^	Plasma from venous blood	LC-MS/MS (20)
		Phase 2, Coartem 480 mg twice daily for 3 days (at 0, 8, and 24 h; thereafter every 12 h) taken with 40 ml of soy milk (0.8 g fat) and, for all doses except the second, a meal containing 6 g of fat within 1 h of each dose	0, 0.5, 1, 1.5, 2, 3, 4, 5, 6, 8, 14, 24, 30, 36, 42, 48, 54, 60, 61.5, 62, 63, 64, 65, 66, 68, 70, 72, 96, 120, 144, 168, 336, and 504 h after the 1st dose	Plasma from venous blood	LC-MS/MS (20)
InterACT (53)	Tanzania	480 mg Coartem twice daily for three days, taken with yogurt	Days 3, 7, 14, 28, and 42 after the 1st dose	Plasma from venous blood	LC-MS/MS (20)
Uganda study 1 (9)	Uganda	480 mg Coartem, single dose taken with standard Ugandan breakfast	0, 1, 2, 4, 6, 8, 12, 24, 48, and 72 h after the 1st (single) dose	Plasma from venous blood	LC-MS/MS (25)
Uganda study 2 (17)	Uganda	480 mg Coartem twice daily for 3 days taken with standard Ugandan breakfast	0, 1, 2, 4, 8, 12, 24, 48, 72, 96, and 120 h after the 6th (and last) dose	Plasma from venous blood	LC-MS/MS (25)
Nigeria study 1 (14)	Nigeria	480 mg Coartem twice daily for 3 days with standard Nigerian meal 30–60 min postdose	0, 0.5, 1, 1.5, 2, 3, 4, 6, 8, 10, 12, 24, 48, 72, and 96 h after the 6th (and last) dose	Plasma from venous blood	HPLC (50)
Nigeria study 2 (18)	Nigeria	480 mg Coartem twice daily for 3 days with advice to eat before medication	Day 7 after the 1st dose	Capillary whole blood from a ﬁnger prick spotted on a dried blood spot	LC-MS/MS (1,000)
U.S. healthy volunteer study (8)	USA	480 mg Coartem twice daily for 3 days with advice to take all drugs with a meal	0, 0.5, 1, 2, 4, 6, 8, 12, 24, 48, 72, 96, 120, 168, 216, and 264 h after the 6th (and last) dose	Plasma from venous blood	LC-MS/MS (1.43)
Uganda study 3 (29)	Uganda	480 mg Coartem twice daily for 3 days taken with standard Ugandan breakfast	0, 1, 2, 4, 8, 12, 24, 48, 72, 96, 120, 192, 480, and 600 h after the 6th (and last) dose	Plasma from venous blood	LC-MS/MS (25)
Uganda study 4 (20)	Uganda	480 mg Coartem twice daily for 3 days taken with standard Ugandan breakfast	0, 1, 2, 4, 8, 12, 24, 48, 72, 96, 168, and 264 h after the 6th (and last) dose	Plasma from venous blood	LC-MS/MS (25)

a LC-MS/MS, liquid chromatography-tandem mass spectrometry; HPLC, high-performance liquid chromatography; LLOQ, lower limit of quantification.

b The samples from 61.5 to 70 h were not drawn for the single-dose phase of SEACAT 2.4.2.

### Population pharmacokinetics of lumefantrine.

**FIG 1:**
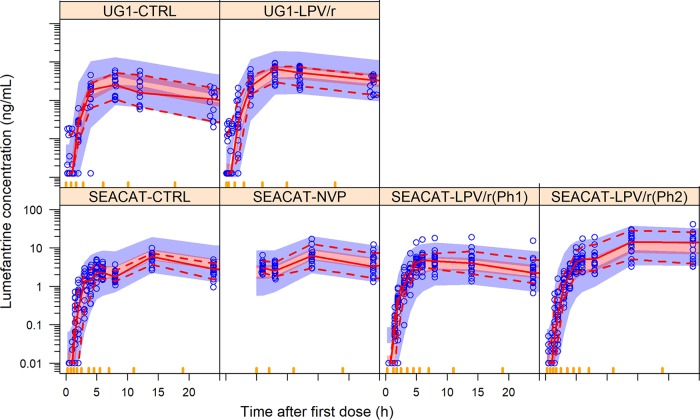

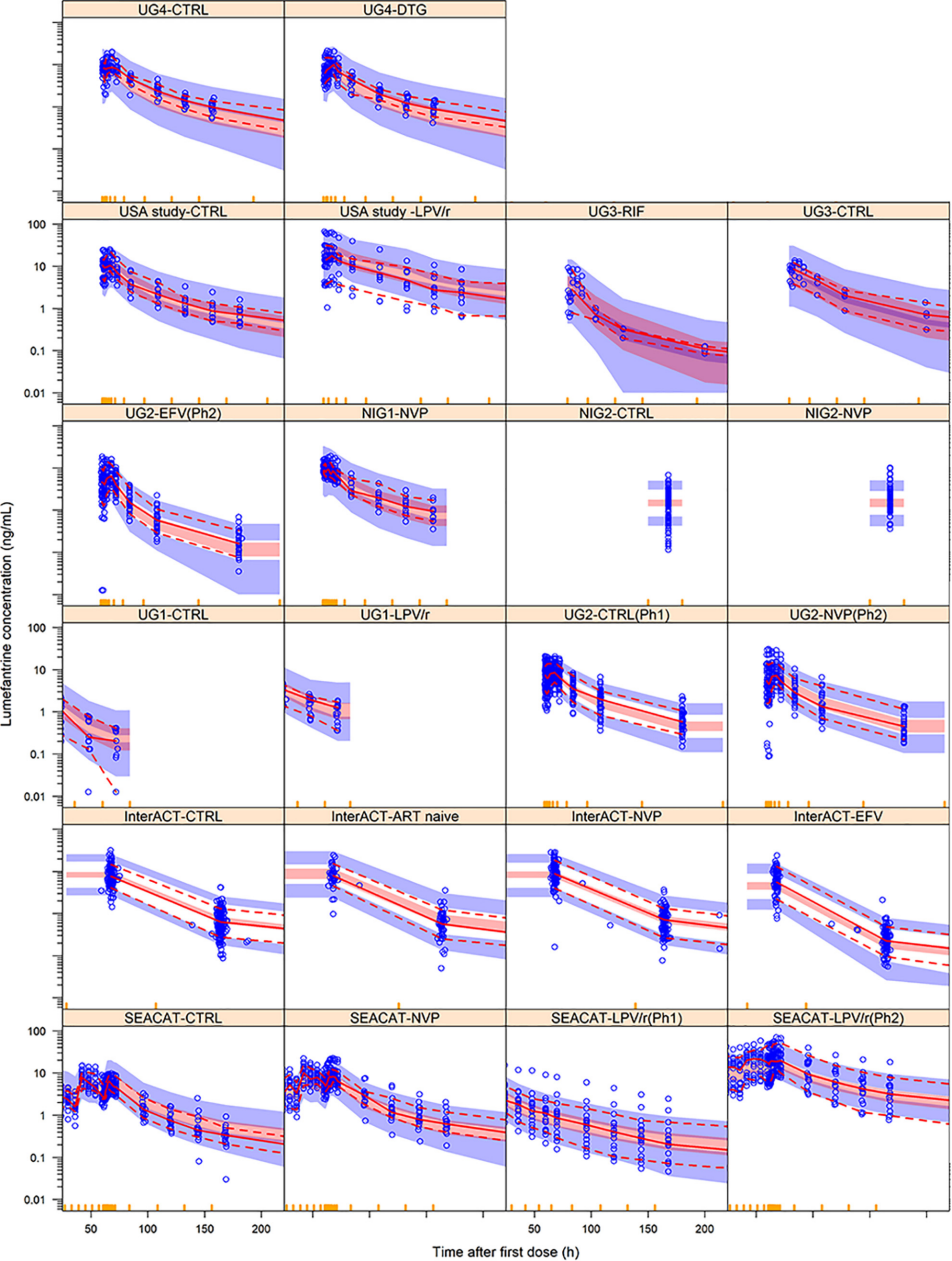

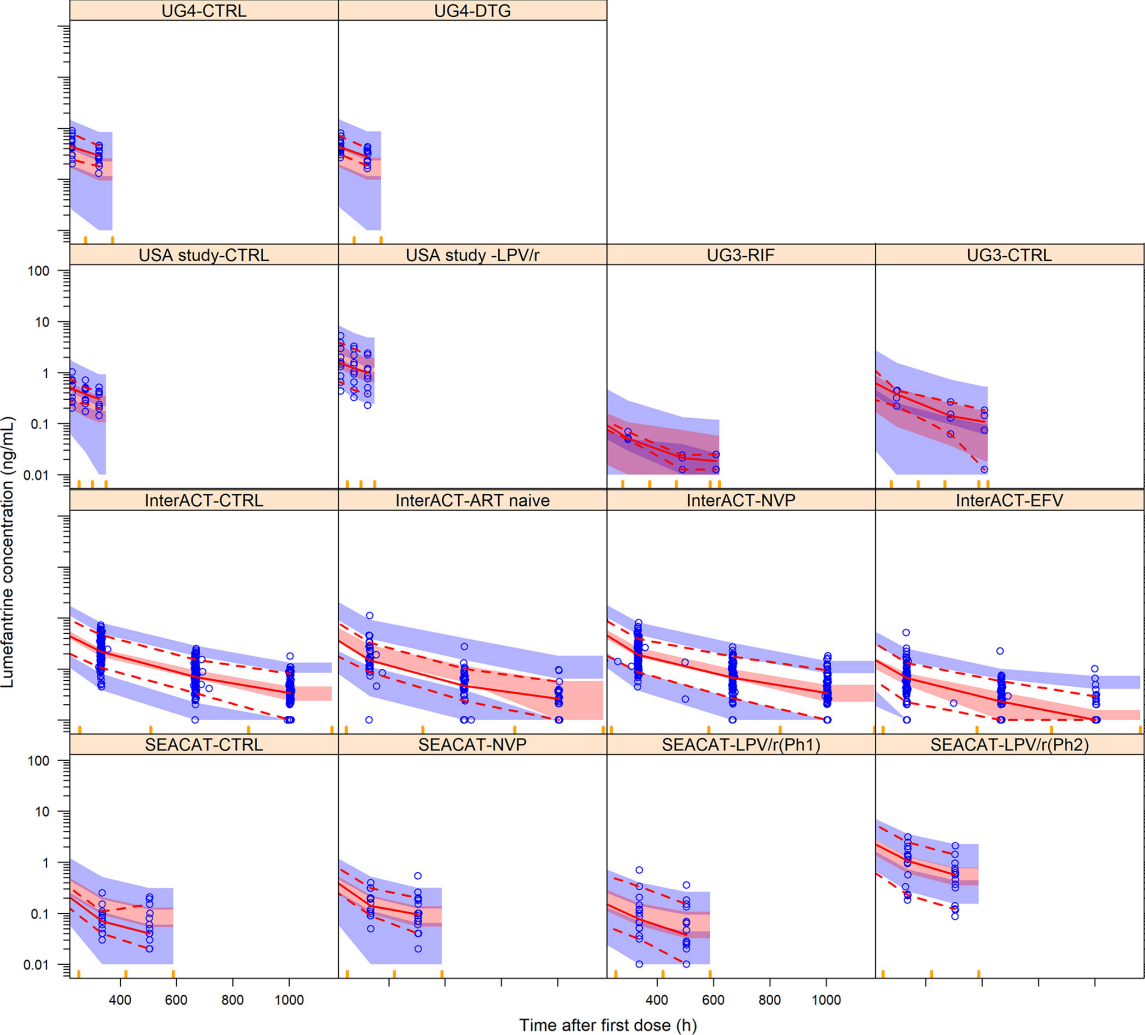
Visual predictive check of lumefantrine concentrations versus time, stratified by study and treatment arm. CTRL, control arm; Ph, phase. The observed lumefantrine concentrations (in the log scale) versus time after the first dose are displayed as blue dots. The solid and dashed lines are the 50th, 5th, and 95th percentiles of the observed plasma concentration, while the shaded areas are the 90% confidence intervals for the same percentiles, as predicted by the model. The three panels correspond to the data collected from 0 to 24 h, 25 to 220 h, and 221 to 1,175 h after the first dose, respectively.

**(i) Structural model and effect of body size.** The population pharmacokinetics of lumefantrine was best described with a three-compartment disposition model (objective function value reduction [ΔOFV] of 930 points when comparing to a two-compartment model; *P* < 0.001) with first-order elimination and transit compartment absorption (ΔOFV, 2,616 compared to a more traditional first-order absorption; *P* < 0.001). Final pharmacokinetic parameter estimates are presented in Table 3, and a visual predictive check (VPC) stratified by study and treatment arm is provided in Fig. 1, showing an adequate model fit to clinical data. Allometric scaling using total body weight was included in the model for all disposition parameters to adjust for differences in body size. The use of fat-free mass or normal fat mass as alternative body size descriptors did not improve the model fit significantly compared to the use of total body weight (21). In a typical patient weighing 57 kg, the apparent clearance (CL) was 3.28 liters/h (95% confidence interval [CI], 3.14 to 3.46) (Table 3).

**TABLE 3:** Final lumefantrine population pharmacokinetics parameter estimates

Parameter	Typical value	95% CI^*a*^	BSV, BVV, or BOV (CV%)^*b*^	95% CI^*a*^
Clearance (CL) (liters/h)^*c*^	3.28	3.14 to 3.46	20.8**^+^**, 15.4**^++^**	18.1 to 23.1, 13.3 to 17.5
Central vol of distribution (liters)^*c*^	60	56.3 to 63.9		
Relative oral bioavailability (F)	1 FIXED		30.2**^+^**, 56.5**^+++^**	25.4 to 35.6, 53.8 to 58.9
Mean absorption transit time (h)	2.86	2.74 to 2.94	31.9**^+++^**	28.8 to 35.1
No. of hypothetical transit compartments	7.58	7.06 to 8.10		
First-order absorption rate constant (1/h) (*K_a_*)	0.727	0.62 to 0.83	73.8**^+++^**	67.5 to 80.8
Intercompartmental clearance between central and first peripheral compartment (liters/h)^*c*^	0.63	0.60 to 0.67	29.1**^+^**	26.1 to 32.4
Vol. of distribution of the first peripheral compartment (liters)^*c*^	182	171 to 195		
Intercompartmental clearance between central and second peripheral compartment (liters/h)^*c*^	1.55	1.43 to 1.72		
Vol. of distribution of the second peripheral compartment (liters)^*c*^	39.1	37.1 to 41.2		
Additive error (ng/ml)	32.9	32.2 to 33.5		
Proportional error (%)	14.2	13.9 to 14.4		
Efavirenz on CL (%)	89.9	81.1 to 99.7		
Lopinavir-ritonavir on CL (%)	−50.1	−53.0 to −46.4		
Lopinavir-ritonavir on F (%)	67.2	49.1 to 88.9		
Lopinavir-ritonavir on *K_a_* (%)	−47.6	−56.5 to −37.4		
Rifampin-based TB treatment on CL (%)	142	111 to 180		
First dose in SECAT on F (%)	−48.6	−54.9 to −41.7		
Consecutive morning doses in SEACAT on F (%)	−77.2	−80.7 to −73.8		
Uganda studies on F (%)	−26.9	−32.3 to −20.7		
Nigeria study 1 on F (%)	−60.8	−73.2 to −47.3		
Delay for unobserved dose (h)^*d*^	4.30	2.84 to 5.73		
Scaling factor for DBS concn (fold)^*e*^	2.28	2.05 to 2.55		

a 95% CI of parameter estimates computed with sampling importance resampling (SIR) on the final model.

b +, BSV, between-subject variability; ++, BVV, between-visit variability; +++, BOV, between-occasion variability. All are expressed as approximate coefficient of variation (CV%).

c The typical values of all clearances and volumes of distribution were allometrically scaled with body weight, and the typical values reported are for a patient with a body weight of 57 kg.

d This delay in absorption/dosing time applies to the unobserved dose prior to the pharmacokinetic sampling visit in the Nigeria study 1 and healthy volunteer study in the United States.

e Scaling factor adjusting for the difference between concentrations from DBS (Nigeria study 2) and plasma (all other studies).

**(ii) Drug-drug interactions.** Coadministration of lopinavir-ritonavir-based ART increased lumefantrine exposure substantially; the area under the curve (AUC) was nearly 3.4-fold higher due to 50.1% slower clearance (ΔOFV, 220; *P* < 0.001) and 67.2% increased bioavailability (ΔOFV, 40; *P* < 0.001). Lopinavir-ritonavir-based ART was also found to slow down the rate of absorption by 47.6% (ΔOFV, 33; *P* < 0.001). Efavirenz-based ART significantly increased the clearance of lumefantrine by 89.9% (ΔOFV, 308; *P* < 0.001), thus resulting in 47% lower AUC. Rifampin-based antituberculosis treatment increased lumefantrine clearance by 142% (ΔOFV, 87; *P* < 0.001), thus reducing AUC by 59%. A small number of patients (*n* = 4) were administered both rifampin and efavirenz, and there was a trend toward an even higher clearance of lumefantrine, but this was not statistically significant and was not retained in the final model. Dolutegravir-based ART did not alter lumefantrine exposure. Discordant trends toward slightly higher or lower exposure in the nevirapine-based ART arms were found in the different studies, but no significant effect was found in the combined data after adjusting for other factors (below).

**(iii) HIV and malaria disease effects.** After adjusting for the substantial drug-drug interactions above (and other effects explained below), the IPD meta-analysis also tested for any malaria and HIV disease effects, but none was identified. The HIV-positive (HIV^+^) but ART-naive participants from the SEACAT (South East African Combination Anti-malarial Therapy) study showed a trend toward moderately increased clearance of lumefantrine, but the same trend was not found with the HIV^+^ ART-naive participants in the other studies. As the magnitude of this effect was small and not consistent across studies, it was not retained in the final model. Similarly, no significant consistent difference in pharmacokinetic parameters was found that could be ascribed to malaria infection.

**(iv) Study and other covariate effects.** *(a) Diurnal variation*. After adjusting for the effects described above, significant differences in drug concentrations remained between the studies and, when data were available, between profiles collected after morning or evening doses. These differences were well captured in the model using categorical covariate effects on relative bioavailability (i.e., separate values of bioavailability on specific dosing occasions). The highest bioavailability was observed in the InterACT (Interactions Between Artemether-lumefantrine and Antiretrovirals in HIV-patients With Uncomplicated Malaria in Tanzania) and the SEACAT studies for the evening doses (with no significant difference between these two studies), and this was chosen as the reference value (fixed to 1) to which the bioavailability of other doses was compared. In the SEACAT studies, the relative bioavailability was 48.6% lower for the first (morning) dose (ΔOFV, 63; *P* < 0.001) and 77.2% lower for the consecutive morning doses (ΔOFV, 280; *P* < 0.001). The value of relative bioavailability in the Ugandan studies was similar and was found to be 26.9% lower than the reference (ΔOFV, 31; *P* < 0.001), while the value was 60.1% lower than the reference (ΔOFV, 18; *P* < 0.001) for the 6th (morning) AL dose in the Nigeria study 1. Lumefantrine bioavailability in the U.S. healthy volunteer study was not significantly different from the reference group.

*(b) Matrix effect*. Lumefantrine concentrations in both arms of Nigeria study 2, which was the only study measuring concentrations from whole-blood samples (as opposed to venous plasma samples), were much higher than in all other studies. A scaling factor of 2.28-fold was included to account for this matrix effect (ΔOFV, 122; *P* < 0.001), which is consistent with previous reports (22, 23).

*(c) Dosing time*. The predose (i.e., morning samples before the 6th dose) concentrations in Nigeria study 1 and the U.S. healthy volunteer study were higher and inconsistent with the profile collected after the observed 6th dose. The actual dosing time of the previous (5th) dose was not reported, so it had been imputed to exactly 12 h before the morning dose. We adjusted for this by estimating a delay in the absorption for this specific occasion, ∼4.3 h (ΔOFV, 17; *P* < 0.001).

*(d) Weight-adjusted dose.* Finally, a negative trend between bioavailability and milligrams per kilogram of dose was detected in the ART-naive arms of SEACAT, InterACT, and for the AL-only arm of Uganda clinical study 1. However, this trend was not present in the other studies and arms and was not significant in the model when tested overall.

**FIG 2:**
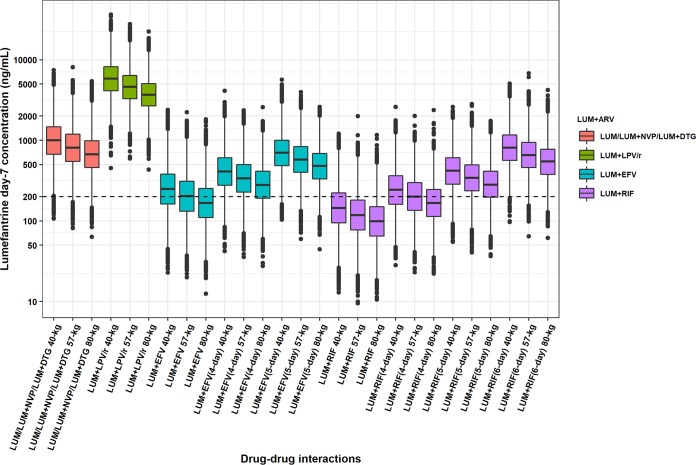
Simulated day 7 concentration of lumefantrine with various drug-drug interactions in the analysis. The box represents the 25th to 75th percentiles, and the whiskers represent the 2.5th to 97.5th percentiles of the simulated day 7 concentration after Monte Carlo simulations (*n* = 10,000). The dashed line at 200 ng/ml denotes the suggested threshold.

**(v) Simulations on the attainment of therapeutic day 7 concentrations.** The Monte Carlo simulations from the final model (Table 4 and Fig. 2) show how body weight and different cotreatments for HIV and tuberculosis affect lumefantrine concentrations and the probability of achieving the purported therapeutic concentration threshold of ≥ 200 ng/ml (22). A typical 57-kg participant (median body weight in the study) is predicted to achieve satisfactory day 7 concentrations when treated with AL alone or concomitantly with nevirapine or dolutegravir and largely exceed them if on lopinavir-ritonavir. On the other hand, the same patient has 49% and 80% probability of not achieving day 7 concentration above the target when cotreated with efavirenz or rifampin, respectively. Additionally, participants with larger body weights are predicted to have lower exposures. The effect of body size on target attainment is modest when AL is used alone or with nevirapine or dolutegravir, but it becomes critical for participants of larger weight cotreated with efavirenz or rifampin. Our model predicts that the risk of day 7 concentrations below the target increases to 62% and 87% for an 80-kg patient on efavirenz or rifampin, respectively. The use of a 4-day regimen of AL is predicted to reduce the risk of subtherapeutic day 7 concentration to 18% and 50% for a typical 57-kg patient on efavirenz or rifampin, respectively, and these probabilities drop further to 3% and 16% with a 5-day regimen. Simulations showed that a 6-day regimen of AL was necessary to reduce the risk of subtherapeutic day 7 concentration to 2% for a typical 57-kg patient on rifampin. For an 80-kg patient, a longer 5-day and 6-day regimen for efavirenz or rifampin cotreatment, respectively, reduces the risk of subtherapeutic concentrations to 5% and 3%.

**TABLE 4:** Simulated day 7 concentration of lumefantrine with various drug-drug interactions in the analysis

Typical body wt of patient (kg)^*a*^	Concn (median [IQR]) (ng/ml) (%)^*b*^								
	Lumefantrine alone or with nevirapine-dolutegravir (3-day regimen of AL)	Lumefantrine plus lopinavir-ritonavir (3-day regimen of AL)	Lumefantrine plus efavirenz			Lumefantrine plus rifampin			
			3-day regimen of AL	4-day regimen of AL	5-day regimen of AL	3-day regimen of AL	4-day regimen of AL	5-day regimen of AL	6-day regimen of AL
40	999 (670–1,476) (0)	5,823 (4,123–8,186) (0)	250 (162–382) (37)	410 (276–606) (11)	700 (483–1,003) (1)	144 (94–222) (69)	243 (161–362) (38)	419 (286–610) (10)	812 (563–1,169) (0)
57	811 (548–1,194) (1)	4,626 (3,294–6,407) (0)	203 (132–310) (49)	336 (228–498) (18)	576 (401–836) (3)	118 (77–182) (80)	201 (135–299) (50)	343 (236–497) (16)	656 (459–941) (2)
80	671 (459–987) (2)	3,686 (2,669–5,058) (0)	166 (110–253) (62)	279 (191–414) (28)	479 (332–684) (5)	99 (65–151) (87)	166 (113–246) (62)	282 (197–413) (26)	547 (378–776) (3)

a 57 kg was the median weight in the study.

b Percentages in parentheses refer to the percentage of individuals below the day 7 threshold of 200 ng/ml after simulations (*n* = 10,000).

## DISCUSSION

In this IPD meta-analysis of lumefantrine pharmacokinetics, we quantified the effect of commonly prescribed ARTs on lumefantrine exposure. To the best of our knowledge, this is the largest IPD meta-analysis of drug-drug interactions of lumefantrine with antiretrovirals to date, combining 6,100 concentrations from 793 adults in 10 studies, 9 from sub-Saharan Africa and 1 from North America. Although most included studies were from Africa, they were carried out in different regions, and the continent is known for its genetic diversity (24), so we believe that significant genetic differences are represented in our pooled analysis. The pooling of individual participant data allowed us to reevaluate and characterize the various drug-drug interactions and other covariate effects more robustly and reliably than in any single study. This was accomplished thanks to the larger sample size and to the flexibility of population pharmacokinetic modeling, which is able to adjust for study-specific differences and the known effects such as patient body size, and separately investigate the drug-drug interactions and disease effects across the different studies. The pooling of data from different studies allowed us to investigate the effect of malaria and HIV infection on lumefantrine exposure and, reassuringly, no effect was found.

The primary aim of this pooled analysis was to characterize the effect of lopinavir-ritonavir-, efavirenz-, dolutegravir-, and nevirapine-based ART on lumefantrine exposure. Lumefantrine exposure is a key determinant of artemether-lumefantrine treatment success, so increased exposure might be associated with a higher cure rate and/or a longer posttreatment prophylactic period (25, 26), while a decrease in concentrations increases the risk of treatment failure.

Lopinavir-ritonavir-based ART increased the exposure of lumefantrine by 3.4-fold and slowed absorption, delaying the time of peak concentration. Lopinavir-ritonavir is a potent inhibitor of CYP3A4 isoenzyme, which explains the increased exposure of lumefantrine when it is coadministered, confirming previous findings from individual studies (8–10, 16). The safety of this increased lumefantrine exposure with lopinavir-ritonavir-based ART is reported in detail elsewhere; in short, there were no serious adverse events and no clinically significant safety concerns raised (10, 26, 27).

Efavirenz-based ART increased the clearance of lumefantrine, thus decreasing exposure by 47%, confirming previous reports and the known effect of efavirenz as a potent inducer of CYP3A4 (11, 12, 17, 26). The findings from this pooled analysis are consistent across studies and provide a more robust estimate since they are based on a larger number of participants and study settings, and they were able to demonstrate the particular importance of this interaction in larger adults.

Rifampin-based tuberculosis treatment was found to decrease lumefantrine exposure by 59%, which was expected, since rifampin is known to be a potent inducer of CYP3A4, and previous physiologically based pharmacokinetic modeling had predicted this *in silico* (28). Clinically, the interaction has been shown in a small study in HIV-infected malaria-uninfected adults (29), which, in this analysis, was pooled with data from patients on rifampin cotreatment from InterACT, thus confirming and making the result more robust. Of the 13 participants on rifampin included in our analysis, four were cotreated with efavirenz, while the rest were not on ART. There was a nonstatistically significant trend toward an even stronger effect on clearance when both efavirenz and rifampin were combined, but the limited sample size limited our ability to robustly characterize this interaction, and this was not included in the final model. Further studies are needed to accurately characterize this clinically significant interaction.

Dolutegravir-based ART had no significant effect on lumefantrine exposure, which suggests that standard doses of AL can be coadministered safely. This finding is of importance considering the rapid adoption of DTG-based ART as a first-line treatment.

Nevirapine-based ART had no significant interaction with lumefantrine in our IPD meta-analysis overall. Nevirapine is reported to be an inducer of CYP3A4 isoenzyme, but the extent of induction is generally considered to be smaller than that of efavirenz (7, 12), with some studies, such as Mouly et al. (30), reporting no induction of nevirapine on CYP3A4 enzymes. Previous reports show inconsistent results regarding the effect of nevirapine on lumefantrine exposure, including the studies contributed to this pooled analysis, but the magnitude of any interaction shown was relatively small. Noncompartmental analyses in Uganda study 2 and Nigeria study 1 reported reduced lumefantrine exposure with nevirapine. The SEACAT study showed that nevirapine-based ART causes a moderate increase in lumefantrine bioavailability (36%), with similar findings in Nigeria study 2. The difference in findings in the IPD meta-analysis with and between individual previous studies may be explained by their including effects of between-occasion variability on bioavailability, while our IPD meta-analysis could identify and characterize the effect of food (fat) coadministration increasing lumefantrine exposure. Accounting for these potential differences between the studies on lumefantrine exposure was necessary for better characterization of the drug-drug interactions. After adjusting for this biologically plausible effect, no effect of nevirapine-based ART on lumefantrine exposure was found in the IPD meta-analysis. This also points to the importance of standardizing food coadministration when lumefantrine pharmacokinetics is investigated.

Simulations from the model helped us to identify participants who are at the highest risk of subtherapeutic concentrations. Most participants treated with AL alone or in combination with nevirapine are predicted to achieve day 7 concentrations above the therapeutic target of 200 ng/ml. However, larger participants are at a relatively higher risk of subtherapeutic day 7 concentrations, e.g., an 80-kg patient on the standard regimen of AL alone or with nevirapine has 2% risk of day 7 concentrations below the target. This finding is in line with previous studies reporting that participants with body weight of ≤65 kg had a better therapeutic outcome compared to those who weighed >65 kg (31, 32). In this IPD meta-analysis, the effect of body size was successfully described using allometric scaling, and no difference in pharmacokinetic parameters remained between participants ≤65 kg and >65 kg. The increase in concentrations due to lopinavir-ritonavir-based ART resulted in day 7 levels well above the target in all participants. However, a significant proportion of participants cotreated with efavirenz or rifampin have day 7 concentrations below 200 ng/ml, and this risk is exacerbated in participants with larger body weight.

As demonstrated in previous studies, the exposure of artemether, the companion drug of lumefantrine, was lowered by concomitant administration of efavirenz or rifampin, but not with lopinavir-ritonavir (11, 12, 26). This may further increase the risk of artemether-lumefantrine treatment failure in those on concomitant efavirenz or rifampin and may hasten the development of artemisinin and/or lumefantrine resistance. Hyperparasitemia is another important risk factor for artemether-lumefantrine treatment failure (22). Drug interactions with efavirenz and rifampin, particularly in participants with other risk factors such as large body weight or hyperparasitemia, are of particular concern given the high prevalence of molecular markers *mdr*86N and *crt*76K associated with reduced lumefantrine susceptibility (33–35) and that artemisinin resistance has been confirmed in at least six countries in Southeast Asia (36, 37).

Alternative dosing regimens for AL are needed to balance the effect of these drug-drug interactions and ensure successful therapeutic outcomes. Unfortunately, simply increasing the number of tablets administered at each dose is precluded by lumefantrine absorption being readily saturable and dose limited with the currently available formulations (16). The use of new formulations as proposed by Jain et al. may present a valuable alternative to circumvent this decreased exposure of lumefantrine (38). A recent study by Tun et al. and Onyamboko et al. where the standard 3-day course of AL was compared to the extended 5-day regimen reported that the extended regimen was well tolerated (39, 40). The simulations from this analysis predict that an extended 5- or 6-day AL regimen overcomes the effect of drug-drug interactions with efavirenz and rifampin, respectively, reducing the chances of subtherapeutic concentrations from ≥60% to under 5%, even for an 80-kg person. Prospective clinical drug-drug interaction studies are needed to evaluate whether these extended regimens of AL or a new lumefantrine formulation can compensate adequately for the effects of interacting drugs such as efavirenz and rifampin.

### Limitations.

The pooling of data from diverse studies also presented some challenges in the IPD meta-analysis. We adjusted the differences between the studies and occasions according to the available information on food intake with the dose, but the influence of any undocumented confounding factors cannot be excluded, as is always the case in pooled analyses. Further studies are recommended to accurately quantify the effect of concomitant food coadministration and diurnal variation on lumefantrine exposure. The uncertainty in the time of dosing history for the Nigeria study 1 and the U.S. healthy volunteer study and the different matrix of drug concentration measurement in Nigeria study 2 were the other hurdles faced. However, the inclusion in the model of the estimation of time of the previous dose and a matrix scaling factor have mitigated the consequences of this uncertainty and allowed us to include these two data sets in our analysis.

### Conclusion.

A model-based IPD meta-analysis was performed to describe the population pharmacokinetics of lumefantrine using data from multiple studies and robustly characterize drug-drug interactions between lumefantrine and commonly used antiretroviral drugs. No significant effect of nevirapine- and dolutegravir-based ART coadministration, malaria, or HIV disease were found. Lopinavir-ritonavir-based ART dramatically increased lumefantrine exposure, while efavirenz-based ART and rifampin-based antituberculosis treatment significantly reduced lumefantrine exposure significantly, particularly in large adults. This warrants further prospective investigation to inform dose modifications given that lumefantrine absorption is readily saturable and dose limited. Various approaches, such as extended 5- or 6-day regimens of AL for participants on efavirenz-based ART or rifampin-based antituberculosis treatment or new formulations of lumefantrine, need to be evaluated to ensure optimal artemether-lumefantrine treatment response. In the interim, full adherence to AL administered with dietary fat and closer monitoring of treatment response is required in these participants.

## MATERIALS AND METHODS

### Data acquisition.

A search was conducted in PubMed, EMBASE, clinicaltrials.gov, Google Scholar, various conference proceedings, and in the Worldwide Antimalarial Resistance Network (WWARN) pharmacology publication database to identify relevant antimalarial clinical studies published between 1990 and 2016. The search strategy used key terms “lumefantrine pharmacokinetics” or “lumefantrine concentration,” “clinical study,” and “HIV” or “antiretroviral.” Inclusion criteria permitted data sets of participants treated with at least one dose of artemether-lumefantrine with or at risk of malaria or healthy volunteers who were HIV infected or uninfected and/or treated with antiretroviral(s), and with at least one or more postdose concentrations of lumefantrine (plus desbutyl-lumefantrine) measured. Under the auspices of the WWARN, corresponding authors of relevant studies were invited to participate in this IPD meta-analysis (https://www.wwarn.org/working-together/study-groups/artemether-lumefantrine-arv-pk-study-group). WWARN is a collaborative data-sharing platform which provides an opportunity to share data and results from studies in the field of antimalarial treatment. Participating authors agreed to the WWARN terms of submission (41), which ensure that all data uploaded were anonymized and obtained with informed consent and in accordance with any laws and ethical approvals applicable in the country of origin. The WWARN semiautomated data management, curation, and analysis tools converted submitted data into a set of defined data variables in a standard format, following the WWARN clinical and pharmacology data management and statistical analysis plans (42, 43). Individual study protocols were available for all trials included, either from the publication or as a metafile submitted with the raw data.

### Population pharmacokinetic modeling.

The population pharmacokinetics of lumefantrine was described using nonlinear mixed-effects modeling in the software NONMEM (version 7.4.2) and the algorithm first-order conditional estimation with eta-epsilon interaction (FOCE-I) (44). Various tools such as Perl Speaks NONMEM (PsN version 4.7.12), Pirana, and Xpose were used to aid the model development and to generate model diagnostics (45). R software was used to generate plots and to perform postmodeling analyses (46).

Various structural models were attempted, from one- to three-compartment disposition with 1st-order elimination and 1st-order absorption, testing the inclusion of lag time or a chain of transit compartments to describe the delay in the onset of absorption (47). Both the between-subject variability and between-occasion variability were assumed to be log-normally distributed. Another level of variability, i.e., the between-visit variability (BVV), was introduced to capture the difference between phase 1 and phase 2 in SEACAT 2.4.2 study, Uganda studies, and the healthy volunteer study from the United States. Allometric scaling was used to adjust for the effect of body size on disposition parameters with allometric exponents fixed to 0.75 for clearance parameters and 1 for volumes of distribution (48). Besides total body weight, fat-free mass and normal fat mass were tested as alternative descriptors to characterize the size of drug-clearing organs and blood flows through them and to explore the possibility that lumefantrine may distribute differentially between muscle or fat tissue (21). A combined additive and proportional error model was used to describe residual unexplained variability. All samples with concentrations below the limit of quantification (BLQ) were handled with the M6 method as described by Beal (49), i.e., BLQ samples were replaced with half of the LLOQ value, except for consecutive values in a series where the trailing BLQ values were omitted from the model fit but were included in simulation-based diagnostic plots, such as VPCs. Model development and the inclusion of parameter-covariate relationships was guided by drops in the NONMEM OFV, assumed to be χ^2^ distributed and thus using a 3.84-point drop as significant at a *P* value of < 0.05 for the inclusion of a single parameter in a nested model, inspection of diagnostic plots, including visual predictive checks (50), and considering at each step the physiological and scientific plausibility of the proposed change. The robustness of the parameter estimates of the final model was assessed using the sampling importance resampling (SIR) method (51).

The strategy used for the inclusion of data from every single study into the joint model was based on the one proposed by Svensson et al. (52). The data from each study were first briefly explored separately and included one by one, starting from the SEACAT studies, which were analyzed first, since they provided richly sampled pharmacokinetic profiles and had an accurate recording of time and concentration for all doses. Further studies including stepwise and significant parameter-covariate relationships were explored. If, after testing all the known/observed covariates, a systematic bias could still be seen in the model prediction of the newly added study, a study effect was included to adjust for the unexplained difference and prevent it from skewing the estimates of other covariate effects.

Monte Carlo simulations (*n* = 10,000) based on the final model were used to predict the lumefantrine concentrations achieved with the current dosing recommendations in participants of different body weights and cotreated with different concomitant medications. To evaluate the expected effect of the pharmacokinetic differences on the therapeutic outcome, we calculated the probability of target attainment in the various scenarios using the suggested value of lumefantrine day 7 concentrations above 200 ng/ml, which has previously been associated with better cure rates (22).
